# Ultrasound diagnosis of traumatic pneumothorax

**DOI:** 10.4103/0974-2700.41788

**Published:** 2008

**Authors:** Michael B Stone

**Affiliations:** Department of Emergency Medicine, SUNY Downstate, 450 Clarkson Avenue Box 1228, Brooklyn, NY 11203, USA

## CASE HISTORY

**Figure 1:**
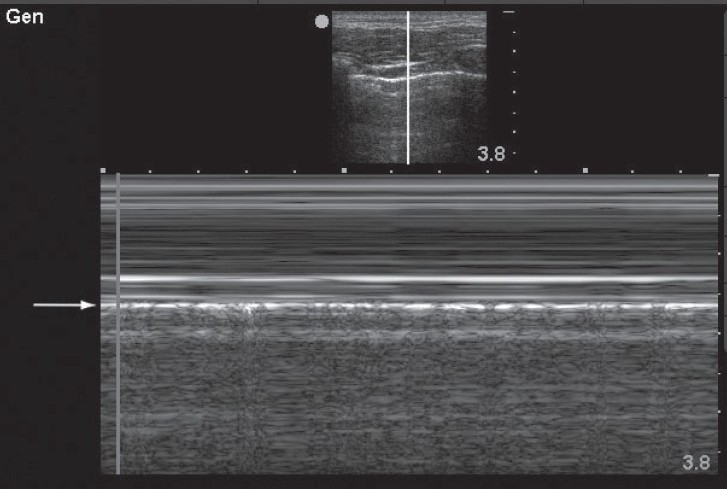
This M-mode image demonstrates a linear, laminar pattern in the tissue superficial to the pleural line (arrow) and a granular or “sandy” appearance deep to the pleural line. This phenomenon, known as the “seashore sign” indicates normal lung sliding and excludes pneumothorax at this interspace

**Figure 2:**
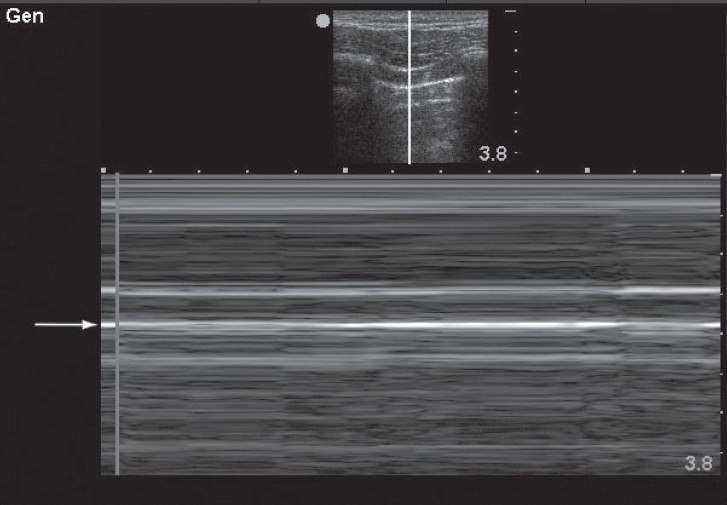
This M-mode image demonstrates a linear, laminar pattern in the tissue superficial to the pleural line (arrow) and a similar linear pattern deep to the pleural line. This phenomenon, known as the “stratosphere sign” or “barcode sign” indicates absent lung sliding and suggests the presence of pneumothorax at this interspace

**Figure 3:**
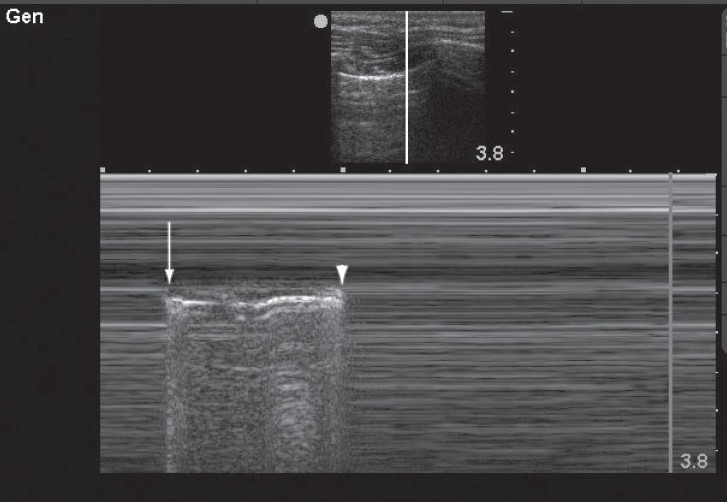
This M-mode image demonstrates an alternating pattern of absent lung sliding with normal lung sliding. This occurs at the boundary of the pneumothorax where during inspiration the lung is seen to slide transiently (arrow) and during expiration lung sliding is abolished (arrowhead) This phenomenon, known as the “lung point,” confirms the presence of pneumothorax

A 36-year-old man presented to our emergency department complaining of right-sided chest pain after a motor vehicle collision. Physical examination revealed bilateral breath sounds and a supine chest radiograph was unremarkable. A 10-5 MHz linear transducer (SonoSite MicroMaxx, Bothell, WA, USA) was used to obtain ultrasound images at the patient's left anterior chest wall [Figure 1], right anterior chest wall [Figure 2] and right lateral chest wall [Figure 3].

## DISCUSSION

*Traumatic pneumothorax*. Shortly after the primary survey the patient developed dyspnea, tachycardia, and hypoxia. A rush of air was encountered upon entering the right pleural space and a 36-French thoracostomy tube was inserted. The patient's symptoms improved and he was admitted to the Trauma service for further management.

Supine chest radiography has low sensitivity for traumatic pneumothorax.[1] Several studies have demonstrated high sensitivity and specificity for thoracic ultrasound for the detection of occult pneumothorax in critical care,[2] and trauma patients.[3] Absent lung sliding suggests pneumothorax, but can occur in the presence of multiple other conditions such as mainstem intubation, acute respiratory distress syndrome, or pleural adhesions. The lung point is an ultrasound sign with 100% specificity for pneumothorax,[4] and can be used to determine the size of the pneumothorax.[5]
